# STAT1 Interacts with RXRα to Upregulate ApoCII Gene Expression in Macrophages

**DOI:** 10.1371/journal.pone.0040463

**Published:** 2012-07-12

**Authors:** Violeta G. Trusca, Irina C. Florea, Dimitris Kardassis, Anca V. Gafencu

**Affiliations:** 1 Institute of Cellular Biology and Pathology, “Nicolae Simionescu” of the Romanian Academy, Bucharest, Romania; 2 Institute of Molecular Biology and Biotechnology, Foundation for Research and Technology of Hellas, Medical School, University of Crete, Heraklion, Crete, Greece; University of Texas Southwestern Medical Center, United States of America

## Abstract

Apolipoprotein CII (apoCII) is a specific activator of lipoprotein lipase and plays an important role in triglyceride metabolism. The aim of our work was to elucidate the regulatory mechanisms involved in apoCII gene modulation in macrophages. Using Chromosome Conformation Capture we demonstrated that multienhancer 2 (ME.2) physically interacts with the apoCII promoter and this interaction facilitates the transcriptional enhancement of the apoCII promoter by the transcription factors bound on ME.2. We revealed that the transcription factor STAT1, previously shown to bind to its specific site on ME.2, is functional for apoCII gene upregulation. We found that siRNA-mediated inhibition of STAT1 gene expression significantly decreased the apoCII levels, while STAT1 overexpression in RAW 264.7 macrophages increased apoCII gene expression. Using transient transfections, DNA pull down and chromatin immunoprecipitation assays, we revealed a novel STAT1 binding site in the −500/−493 region of the apoCII promoter, which mediates apoCII promoter upregulation by STAT1. Interestingly, STAT1 could not exert its upregulatory effect when the RXRα/T3Rβ binding site located on the apoCII promoter was mutated, suggesting physical and functional interactions between these factors. Using GST pull-down and co-immunoprecipitation assays, we demonstrated that STAT1 physically interacts with RXRα. Taken together, these data revealed that STAT1 bound on ME.2 cooperates with RXRα located on apoCII promoter and upregulates apoCII expression only in macrophages, due to the specificity of the long-range interactions between the proximal and distal regulatory elements. Moreover, we showed for the first time that STAT1 and RXRα physically interact to exert their regulatory function.

## Introduction

**Figure 1:**
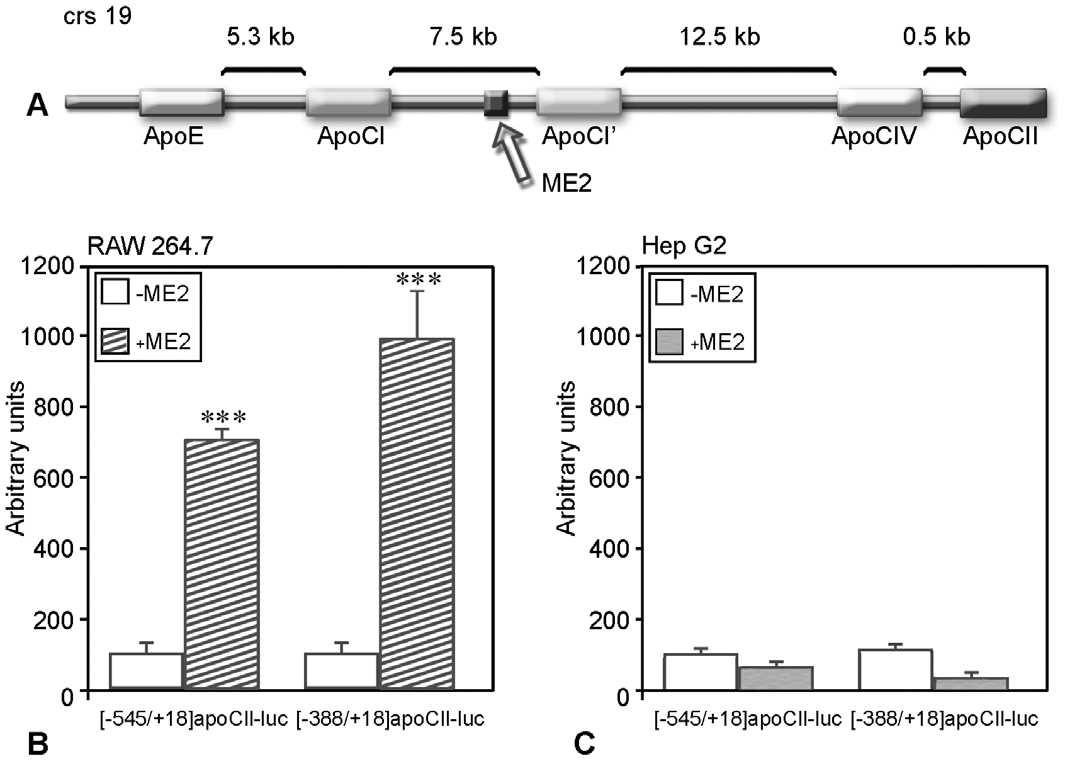
ApoCII promoter activity is upregulated by ME.2 in macrophages but not in hepatocytes. *Panel A.* Schematic representation of the apoE/apoCI/apoCI’/apoCIV/apoCII gene cluster and the location of ME.2. *Panels B and C.* RAW 264.7 macrophages and HepG2 cells were transiently transfected with plasmids in which [−545/+18]apoCII or [−388/+18]apoCII promoter fragments were cloned in the presence (+ME.2) or in the absence (–ME.2) of ME.2 in pGL3 basic vector. In RAW 264.7 macrophages, both promoter fragments activities can be enhanced by ME.2 (Panel B) while in HepG2 hepatocytes, ME.2 cannot increase apoCII promoter activity (Panel C).

Apolipoprotein CII (apoCII) is a 79-amino acid protein that plays an important role in the catabolism of triglyceride-rich lipoproteins [1], [2], being a specific physiological activator of lipoprotein lipase [3], [4]. ApoCII is mainly synthesized and secreted by the liver, as a component of nascent Very Low Density Lipoprotein, but it is also produced to a lesser extent by the intestine as a component of chylomicrons [5], [6]. Other minor synthesis sites are the macrophages infiltrated in the atherosclerotic plaques, the pancreas, the brain and the lung [7]–[8]. Patients with inherited apoCII deficiency are unable to clear triacylglycerol-rich lipoprotein particles from their plasma and develop severe hypertriglyceridaemia [2], [5], [6]. On the other hand, it was shown that transgenic mice overexpressing apoCII develop hypertriglyceridaemia [9]. Thus, normal levels of apoCII are critical for optimal triglyceride metabolism. The regulatory mechanisms of apoCII gene expression that maintain the physiological concentrations of apoCII in the plasma are not fully understood. The human apoCII gene has been mapped on the long arm of chromosome 19 in a gene cluster that contains the apoE, apoCI, apoCI’, apoCIV and apoCII genes [10]–[12]. The region between the apoCIV and apoCII genes is only 545-nucleotides long and represents the proximal promoter of apoCII [1]. So far, few transcription factors have been described to bind to the apoCII promoter, including the nuclear receptors HNF-4, ARP-1, and RXRα/T3Rβ [13], [14]. Cell-specific distal enhancers designated as hepatic control regions (HCRs) and multienhancers (MEs) that regulate the expression of the genes belonging to the apoE/apoCI/apoCI’/apoCIV/apoCII gene cluster, in the liver and macrophages, respectively, were reported [15]–[17]. Studies in transgenic mice established that the hepatic expression of the genes belonging to the above cluster is controlled by HCRs [18], [19]. In addition, it was reported that fibrates (PPARα ligands) decrease hepatic apoCII gene expression in rats [20]. In addition, it was shown that bile acids together with retinoids mediate the induction of the apoCII promoter in human hepatoma HepG2 cells by activating nuclear receptors FXR and RXR, which bind to the HCRs [21]. Multienhancer 1 (ME.1) and multienhancer 2 (ME.2), are both 620 bp in length and are located in the regions between the apoE-apoCI and apoCI-apoCI’ genes, respectively ([17], Fig. 1A). Studies in transgenic mice demonstrated that ME.1 and ME.2 are important for apoE gene expression in macrophages and the brain [17], [22]. So far, two LXR response elements (LXREs) located within the multienhancers ME.1 and ME.2 were shown to be involved in apoCII gene induction by ligands for LXR and RXR [7]. Recently, several data showed the involvement of STAT1 in lipid metabolism and atherosclerosis. We previously showed that a STAT1 binding site present on ME.2 is biologically active and important for apoE gene regulation [23].

The aim of the present work was to elucidate the regulatory mechanisms involved in apoCII gene modulation in macrophages. We show by Chromosome Conformation Capture (3C) assays that in macrophages, ME.2, a DNA fragment located 20.25 kb upstream of apoCII gene, physically interacts with the apoCII promoter. This interaction facilitates the transcriptional enhancement of the apoCII promoter by the transcription factors bound on ME.2. We demonstrate that STAT1 can bind on ME.2 as well as on the apoCII proximal promoter, and transactivate the apoCII promoter. For the first time we reveal that STAT1 cooperates with RXRα for apoCII gene upregulation in macrophages.

## Materials and Methods

### Chemicals

Restriction and modification enzymes were from New England Biolabs (Beverly, MA) and Promega (Madison, WI); DMEM, RPMI and fetal calf serum were from Invitrogen (Life Technologies Inc., Gaithersburg, MD); ECL Western blotting kit from Pierce (Rockford, USA); pGL3 basic vector, Luciferase assay system and phorbol 12-myristate 13-acetate (PMA) were from Promega (Madison, WI); all other chemicals were from Sigma Chemical Co (St. Louis, MO). The primers were obtained from Metabion Inc. (Martinsried, Germany). Lipofectamine 2000, ViraPower Lentiviral Expression System, TRIzol reagent and glutathione-agarose beads were from Invitrogen (Life Technologies Inc., Gaithersburg, MD), siRNA for STAT1, scrambled siRNA and the antibodies were from Santa Cruz (Santa Cruz Biotechnology, CA), iQ SYBR Green Supermix for Real Time PCR Bio-Rad (Hercules, CA).

### Plasmid Constructions

The human apoCII proximal promoter [−545/+18], a deletion mutant [−388/+18], and a mutant in the region [−152/−135] (which affects the RXRα/T3Rβ binding site), cloned into pUCSH-CAT vector, have been described previously [13]. The fragments corresponding to the apoCII promoter were subcloned into the KpnI/XhoI sites in pGL3 basic vector upstream of the luciferase reporter gene (luc), obtaining the following plasmids: [−545/+18]apoCII-luc, [−388/+18]apoCII-luc and [−388/+18]apoCIImut-luc. In addition, ME.2 which was previously cloned in pGL3 vector [23], was inserted upstream of the apoCII promoter or its mutants mentioned above, obtaining the following plasmids: ME.2 [−545/+18]apoCII-luc, ME.2 [−388/+18]apoCII-luc, and ME.2 [−388/+18]apoCIImut-luc.

### Cell Culture

Human monocytes were isolated from blood obtained from healthy donors, and cultured as previously described [23]. Human THP-1 monocytic cells were kindly provided by Dr. D.T. Boumpas (University of Crete Medical School, Heraklion, Greece) and cultured in RPMI as described in [24]. Blood monocytes and THP-1 cells were differentiated into macrophages by exposure to 100 nM PMA, for 48 h. RAW 264.7 mouse macrophages, originally from ATCC, were kindly provided by C. Tsatsanis (University of Crete Medical School, Heraklion, Greece), and cultured as described in [25]. HEK-293T and HepG2 cells, originally from ATCC, were cultured in DMEM as described in [26].

### Transient Transfections

RAW 264.7 and HepG2 cells were transiently transfected using the calcium phosphate co-precipitation method or by lipofection using Lipofectamine 2000. Forty hours after transfection, luciferase and β-galactosidase activity were determined as described [27].

### Chromosome Conformational Capture (3C) Assays

Monocytes-derived macrophages, PMA-activated THP-1 monocytes and HepG2 cells were subjected to 3C assay, as previously described [23]. The ligation products were detected by PCR using the following primers: apoCII +1378 (forward), and ME.2+577 or ME.2 −712 (reverse), described in Table 1.

**Table 1:** The sequences of the primers and oligonucleotides used for cloning, RT-PCR, 3C analysis and DNA pull down assay.

Primers for:	5′-3′ sequence:
**3C analysis**	
apoCII +1378 forward	GCCCTAAGGTAACACATTTGATC
ME.2+577 Reverse	GAAGGGAAGAAGGGGCATACACTC
ME.2 −712 Reverse	GACAACCTCTGCTCCTCTCCACCG
ME.2+19 Forward	GGGGTACCTCACTGAGCCCTACTGGATGTTCTTGGTTG
ME.2+488 Reverse	GGGGTACCCCTGCTTCCCACCATCACCAC
**DNA pull down assay**	
STAT1/CII WT Forward	Biotin -CACTGCCTCCCAGGAATCAATGA
STAT1/CII WT Reverse	TCATTGATTCCTGGGAGGCAGTG
STAT1/CII MUT Forward	Biotin -CACTGCCGCCAATGCCTCAATGA
STAT1/CII MUT Reverse	TCATTGAGGCATTGGCGGCAGTG
**RT-PCR**	
Mouse apoCII Forward	GAGCCAGGATAGTCCCTTCC
Mouse apoCII Reverse	AAAATGCCTGCGTAAGTGCT
Human apoCII Forward	GTCAGCAAAGACAGCCGCCCA
Human apoCII Reverse	AGCCCCTCCATCTTGGCCCTT
GAPDH Forward	ACCACAGTCCATGCCATCAC
GAPDH Reverse	TCCACCACCCTGTTGCTGTA
**ChIP**	
apoCII −738 Forward	TTGAGGGGATGACAAGGAAG
apoCII −336 Reverse	GAACCACTGGGCAACTTAGC
ME.2+140 Forward	TGTGGGATCGGGGAAGCCAGACA
ME.2+440 Reverse	TCCTTCCCCACCAGCTGCCT

### Chromatin Immunoprecipitation (ChIP)

ChIP experiments were performed as previously described [28] using anti-STAT1 antibodies and the immunoprecipitated chromatin was analyzed by PCR using the primers shown in Table 1.

### DNA Pull-down Assays (DNAP)

DNA fragments of the apoCII promoter were biotinylated by PCR, using biotinylated RVprimer3 and GLprimer2 (Promega, Madison WI) and [−545/+18]apoCII-luc or [−388/+18]apoCII-luc plasmids, as templates. The biotinylated PCR products, and the biotinylated oligonucleotides containing the sequence of wild type or mutated [−507/−485]apoCII promoter region (described in Table 1) were immobilized on Dynabeads M-280 Streptavidin. Nuclear extracts were purified from HepG2 or RAW 264.7 cells and incubated with the above biotinylated DNA immobilized on Dynabeads, as previously described [23]. STAT1 bound to the DNA were detected by Western blotting using anti-STAT1 antibodies.

### STAT1 Gene Expression Knock Down

#### STAT1 siRNA(m)

containing a pool of three target specific 19–25 nucleotides was used for STAT1 gene expression inhibition in RAW 264.7 mouse macrophages. The cells were transfected by lipofection with STAT1 siRNA or scrambled siRNA and the obtained samples were subjected to Real Time PCR.

#### shRNA for STAT1

was obtained as we previously described [23]. HepG2 cells were transiently transfected with STAT1 shRNA or scrambled shRNA and the obtained samples were subjected to Real Time PCR.

#### RNAi lentivirus system

Lentiviral particles, were generated using HEK-293T cells which were transfected using Lipofectamine 2000, with plasmids expressing a micro RNA cassette for human STAT1 (pLenti6/miR-hSTAT1, kindly provided by Prof. Yoshihiro Ohmori, Meikai Univ., Saitama, Japan) along with lentivirus packaging vectors (ViraPower Lentiviral Expression System) as described by the manufacturer. PMA-treated THP-1 cells were transduced with the obtained lentiviral particles, and subjected to RT-PCR or Western blotting.

### RT-PCR

Total RNA was extracted from the above transfected cells, as well as from RAW 264.7 macrophages treated with 1 µM 9-cis-retinoic acid (18 h), using TRIzol reagent according to the manufacturer’s instructions. cDNA was synthesized from 1 µg RNA using oligo dT and M-MLV Reverse Transcriptase. The specific primers used are described in Table 1. The fragments obtained by RT-PCR were: 304 bp for mouse apoCII, 365 bp for human apoCII, and 451 bp for GAPDH.

Real Time PCR experiments were performed with iQ SYBR Green Supermix, using 7900 HT Applied Biosystems machine. The reactions were done in a total volume of 10 µl. The Real Time amplifications included 3 min at 95°C, followed by 40 cycles at 95°C for 30 sec, 59°C for 30 sec (for mouse apoCII primers) or 66°C for 30 sec (for human apoCII primers), and 72°C 30 sec. Melting curves were determined at the end of the amplification program. To estimate the relative expression levels, a standard curve, derived from six cDNA dilution series in triplicate was also performed for each experiment. The regression coefficients were greater than 0.99. To normalize the apoCII expression, GAPDH mRNA levels were also tested in similar conditions.

### Western Blotting

The samples were subjected to SDS-PAGE and transferred on nitrocellulose. The blots were tested with anti-STAT1, and anti-actin antibodies, followed by HRP-conjugated secondary antibodies. The protein bands were detected using an ECL kit and LAS-4000 Chemiluminiscent Image Reader (FUJIFILM Europe GmbH, Germany).

### Glutathione S Transferase (GST) Assay

GST-RXRα fusion proteins were expressed in E. coli strain BL21. After stimulation with 1 mM isopropyl-β-D-thiogalactopyranoside for 3 h at 37°C, the bacteria were harvested, resuspended in PBS, sonicated, and lysed in Triton X-100 (1% final concentration). The supernatant obtained after centrifugation at 10,000 rpm, at 4°C for 10 min, was enriched in GST-RXR fusion proteins, as tested by SDS-PAGE and Coomassie Blue staining.

Whole cell extracts were obtained from STAT1-overexpressing RAW 264.7 cells using a hypertonic buffer containing 20 mM Tris pH 7.4, 400 mM KCl, 10% glycerol, 2 mM DTT and protease inhibitors. Pre-equilibrated glutathione-agarose beads were incubated with GST-RXR proteins 18 h at 4°C, and washed 3 times in PBS. Then, GST-RXR immobilized on the beads were incubated with the previously obtained whole cell extracts, in the Interaction Buffer (20 mM Hepes, 5 mM MgCl_2_, 0.2% NP40, 0.2% BSA, 7.5% glycerol and protease inhibitors), for 16 h at 4°C. After coupling, the beads were washed three times with washing buffer (100 mM KCl, 20 mM Hepes pH 7.9, 5 mM MgCl_2_, 0.2% NP40 and protease inhibitors). The bound proteins were eluted in loading buffer and subjected to Western blotting using anti-STAT1 antibodies.

### Co-immunoprecipitation Assay

Cellular extracts were prepared from HEK-293T cells transfected with STAT1 and RXRα expression vectors. The cell extracts were successively incubated with anti-STAT1 or anti-RXRα antibodies (16 h at 4°C) and then with PBS pre-equilibrated protein A/G Sepharose beads (1 h at 4°C). The beads were washed thoroughly, resuspended in reducing loading buffer and subjected to Western blotting using rabbit anti-STAT1 antibodies. As a negative control, we followed the same procedure without the addition of the antibodies or using an antibody specific for other type of molecule (e.g. anti-von Willebrand factor).

#### Statistical analysis of the data

was performed using the one-way analysis variance between groups (ANOVA) from OriginPro 7.5 Software. All values were expressed as means ± SEM. For ‘p’ values less than 0.05, the population means are statistically different.

## Results

### In Macrophages, but not in Hepatocytes, ME2 Upregulates the apoCII Promoter

Previously, it was shown by others and us [7], [23] that in macrophages, ΜE.2 enhances the activity of all promoters of the apoE/apoCI/apoCI’/apoCIV/apoCII gene cluster (schematically described in Fig. 1A). In the present study, we compared the interaction of ME.2 with the apoCII promoter in two cellular types (macrophages and hepatocytes), known to express apoCII. For this purpose, we performed transient transfection experiments in RAW 264.7 macrophages (Fig. 1B) and in HepG2 hepatocytes (Fig. 1C), using plasmids containing the apoCII proximal promoter [−545/+18], or its deletion mutant [−388/+18] cloned upstream of the luciferase gene in the pGL3 basic vector, in the presence (+ME.2) or in the absence (−ME.2) of ME.2. The results demonstrated that in macrophages, the activity of the [−545/+18]apoCII promoter as well as of its deletion mutant [−388/+18] was enhanced by ME.2 (∼7 and ∼8 fold, p<0.002). By contrast, in hepatocytes, ME.2 failed to increase apoCII promoter activity. These data suggested that ME.2 regulates the apoCII promoter in a cell-specific manner.

**Figure 2:**
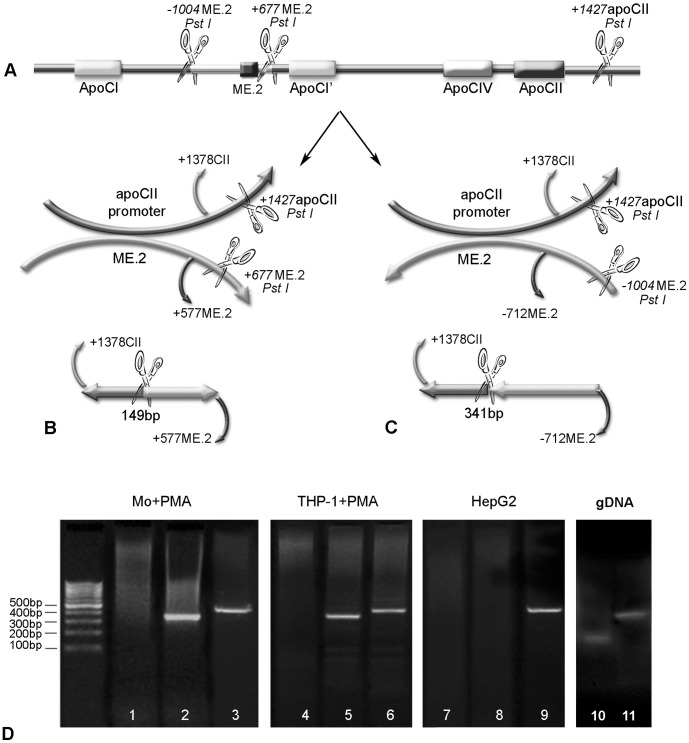
Interactions between apoCII promoter and ME.2 in macrophages as revealed by chromosome conformation capture (3C) assays. *Panels A–C.* Schematic illustration of the linear alignment of ME.2 and apoCII (including the Pst I sites adjacent to these regions) and the possible interactions between apoCII promoter and ME.2 in sense (B) or antisense (C) orientation. For 3C experiments, the PstI restriction enzyme was used. PstI restriction sites are located at positions +1427 in apoCII gene as well as at +677 bp downstream of ME.2 and at −1004 bp upstream of ME.2, as showed in the picture. The primers flanking the newly ligated fragments are also illustrated: for both cases (B and C), the forward primer was +1378CII, and the reverse primers were: +577ME.2 (B) and −712ME.2 (C). The predicted lengths of the ligated fragments are: 149 bp for “sense orientation” (B) and 341 bp and “antisense orientation” interaction with ME.2 (C). *Panel D.* Human peripheral blood monocytes (lanes 1–3) and THP-1 cells (lanes 4–6) differentiated to macrophages by PMA treatment, as well as HepG2 cells (lanes 7–9), were subjected to the 3C assay. PCR fragments amplified with primers +1378CII and +577ME.2 (testing the interaction in sense orientation) are shown in lanes 1, 4 and 7, and PCR products obtained with primers +1378CII and −712ME.2 (testing the interaction in antisense orientation) are shown in lanes 2, 5 and, respectively 8. The positive control for DNA integrity and PCR (amplification of a 468 bp fragment of ME.2) are shown in lanes 3, 6 and respectively 9. ME.2 and the apoCII promoter interact in antisense orientation since only the fragment of 341 bp was obtained from human monocytes and THP-1 cells differentiated with PMA (lane 2 and, respectively 5) whereas no bands were obtained for sense orientation interaction (lanes 1 and, respectively 4). In contrast, in HepG2 cells, 3C experiments gave no bands either for sense or antisense orientation interaction (lanes 7 and, respectively 8), revealing that apoCII promoter and ME.2 do not interact in hepatocytes. As control, genomic DNA isolated from THP-1 cells was used, and the amplification of the ligation products using +1378CII and +577ME.2 primers, and +1378CII and −712ME.2 primers, gave the expected size of the PCR products of 149 bp and 341 bp (Fig. 2D, lanes 10 and 11, respectively).

Physical interactions between the apoCII promoter and ME.2 in macrophages and hepatocytes were tested by Chromosome Conformation Capture (3C) assays. For this purpose, we used human macrophages derived from monocytes isolated from normal blood or THP-1 cells, as well as HepG2 hepatocytes. The linear alignment of ME.2 and apoCII, including the Pst I sites adjacent to these regions (+1427 in apoCII gene, −1004 bp upstream of ME.2 and +677 bp downstream of ME.2) is represented in Fig. 2A. The possible interactions between the apoCII promoter and ME.2 in sense or antisense orientation are illustrated in Fig. 2B and 2C respectively. The location of the PCR primers used in the 3C analysis is also shown. For both cases, the forward primer was +1378CII (starting at +1378 in apoCII gene). The reverse primers were: +577ME.2 (to test the interaction in sense orientation) and −712ME.2 (to test the interaction in antisense orientation). The predicted lengths of the ligated fragments are: 149 bp and 341 bp for sense and antisense orientation interaction with ME.2, respectively (Panel B and C). Our data showed that ME.2 can interact with the apoCII promoter in macrophages, and the interaction takes place in antisense orientation of the two DNA fragments (Fig. 2D, lanes 2 and 5). ME.2 could not interact with the apoCII promoter in the sense orientation (Fig. 2D, lanes 1 and 4). In addition, apoCII promoter and ME.2 could not interact in HepG2 cells in any orientation (Fig. 2D, lanes 7 and 8). In addition, HTB-11 astrocytes, cells that do not express apoCII (tested by RT-PCR) were also employed in 3C experiments and the results were similar with those obtained for HepG2 cells (data not shown). The positive controls for DNA integrity and PCR, showing that equal amounts of DNA template were present in the samples (amplification of a 468 bp fragment of ME.2) are in lanes 3, 6 and 9, respectively. Using genomic DNA isolated from THP-1 cells, we amplified the ligation products having the expected size of 149 bp or 341 bp (Fig. 2D, lanes 10 and 11).

### STAT1 Positively Modulates apoCII Expression in Macrophages, but not in Hepatocytes

**Figure 3:**
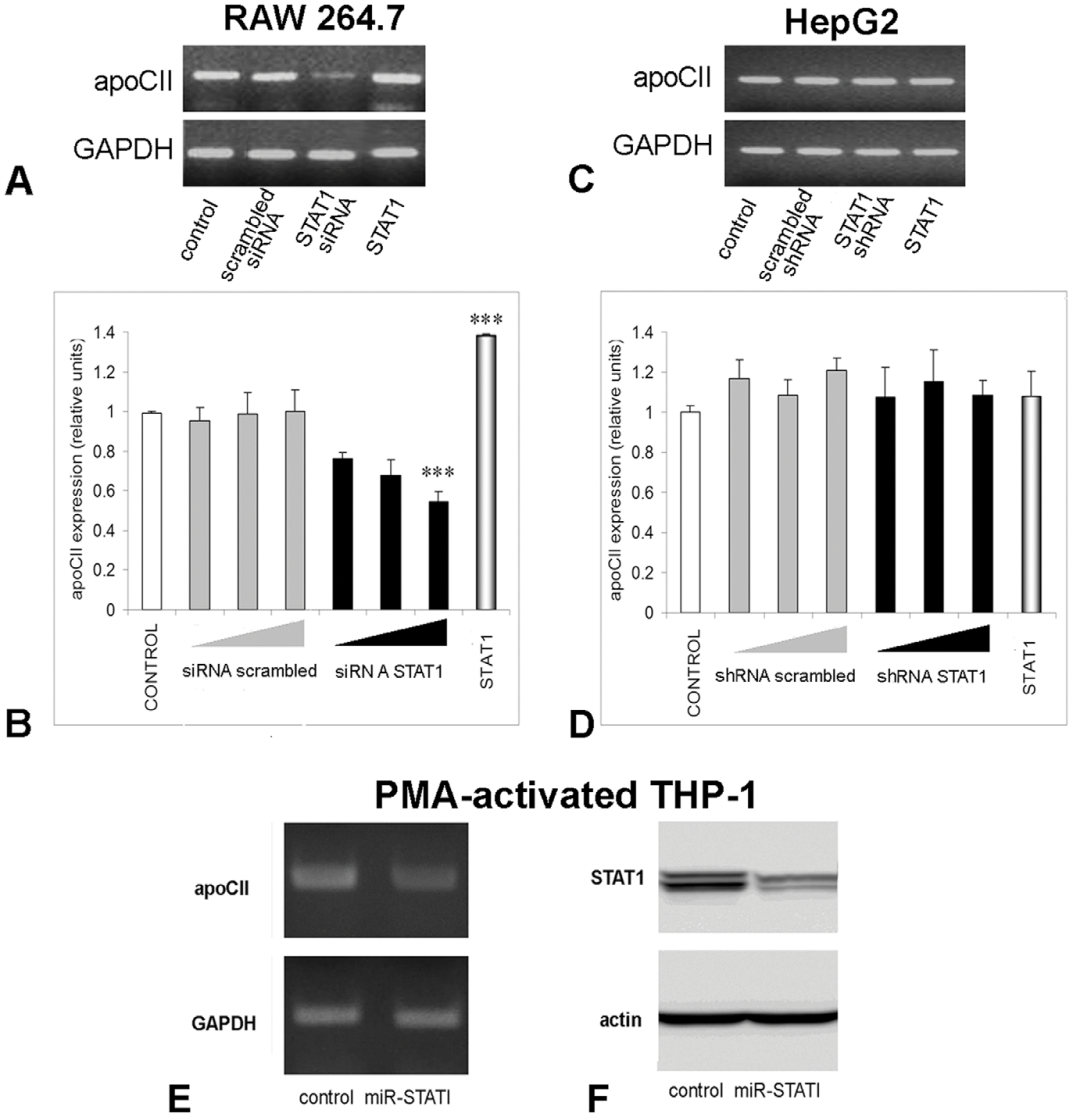
STAT1 positively modulates apoCII expression in macrophages, but not in hepatocytes. To determine apoCII modulation by STAT1 silencing, RAW 264.7 cells were transfected with siRNA specific for mouse STAT1 (*Panel A and B)*, in HepG2 cells were transfected with STAT1 shRNA producing vector (*Panel C and D)*, and in PMA-activated THP-1 cells were transduced with a lentiviral expression vector for a micro RNA cassette for human STAT1 (*Panel E)*. ApoCII modulation was tested by RT-PCR (A, C and E) and quantified by Real Time PCR (B and D). GAPDH expression was tested to normalize the apoCII expression. The results showed that apoCII expression was strongly inhibited in macrophages (Panel A, lane STAT1 siRNA and Panel E lane miR-STAT1), but not in hepatocytes (Panel C, lane STAT1 shRNA). Real Time PCR experiments showed a dose-dependent inhibition of apoCII by STAT1 siRNA in RAW 264.7 macrophages (for the highest dose, p<0.005), but not in HepG2 cells (Panels B, and D, respectively, black columns). As control, we used scrambled siRNA for RAW.264.7 cells, or scrambled shRNA for HepG2 cells, which did not affect significantly the apoCII expression. STAT1 expression was inhibited more than 50% by transduction of the PMA-activated THP1 with miR-STAT1, as detected by Western blotting (Panel F). To determine apoCII modulation by STAT1 overexpression, RAW 264.7 macrophages and HepG2 cells were transfected with a STAT1 expression vector and subjected to RT-PCR. ApoCII mRNA level was increased in STAT1-overexpressing macrophages (Panel A, lane STAT1), but not in hepatocytes (Panel C lane STAT1, and Panel D last column). Real Time PCR experiments revealed an increase of ∼1.4 folds in apoCII expression in STAT1 overexpressing macrophages (Panels B, last columns; p<0.005).

Since we have detected significant STAT1 basal levels in macrophages (RAW 264.7 as well as PMA-activated THP-1 cells), first we tested the effect of STAT1 inhibition on apoCII expression. For this purpose, RAW 264.7 cells were transfected with siRNA specific for mouse STAT1, and PMA-activated THP-1 cells were transduced with a lentiviral expression vector for a micro RNA cassette for human STAT1. As control, we employed HepG2 cells that were transfected with a STAT1 shRNA producing vector. ApoCII modulation was tested by RT-PCR and quantified by Real Time PCR experiments. The data were normalised to GAPDH expression. Data showed a strong inhibition of apoCII expression only in macrophages (Fig. 3A, lane STAT1 siRNA, and Fig. 3E lane miR-STAT1), but not in hepatocytes (Fig. 3C, lane STAT1 shRNA). As control, we used scrambled siRNA for RAW.264.7 cells (Fig. 3A, lane STAT1 scrambled siRNA), or scrambled shRNA for HepG2 cells (Fig. 3C, lane scrambled shRNA). STAT1 inhibition after lentiviral infection was demonstrated by Western blot, using anti-STAT1 antibodies (Fig. 3F).

To quantify the degree of down regulation of apoCII by STAT1 silencing, we performed Real Time PCR. The results showed a dose-dependent inhibition of apoCII by STAT1 siRNA in RAW 264.7 macrophages, but not in HepG2 cells (Figure 3B, and C, respectively, black columns). In control experiments, we used scrambled siRNA or shRNA, which did not affect significantly the apoCII expression in RAW 264.7 and HepG2 cells (Figure 3B, and C, grey columns).

To determine the effect of STAT1 overexpression on apoCII gene expression in RAW 264.7 macrophages and HepG2 cells (as control), the cells were transfected with a STAT1 expression vector and subjected to RT-PCR. Our data showed that apoCII mRNA level was increased in STAT1-overexpressing macrophages, but not in hepatocytes (Fig. 3A, lane STAT1, and Fig. 3C lane STAT1). Real Time PCR experiments revealed an increase of 1.4 folds in apoCII expression in STAT1 overexpressing macrophages, but not in hepatocytes (Figure 3B, and D, last columns). Analysis of the Real Time PCR results reveals that apoCII modulation by STAT1 is statistical significant (p<0.005). This result has a good biological significance since the basal level of STAT1 in RAW 264.7 cells is relatively high, and in STAT1-transfected cells only a double amount of STAT1 was detected by Western Blotting (data not shown).

### STAT1-dependent Transactivation of apoCII Promoter in RAW 264.7 Macrophages

**Figure 4:**
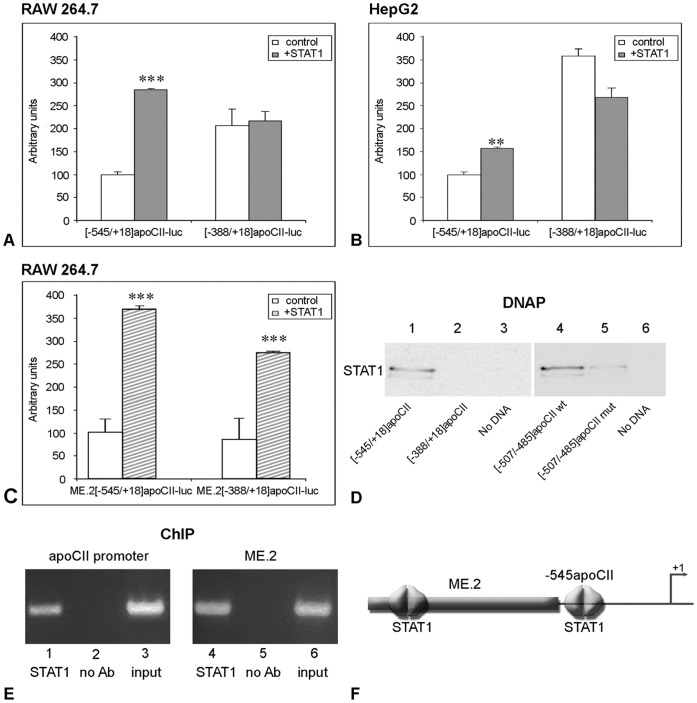
STAT1-dependent transactivation of apoCII promoter in RAW 264.7 cells. *Panel A, B.* RAW 264.7 macrophages (A) and HepG2 hepatocytes (B) were transiently transfected with plasmids containing [−545/+18] or [−388/+18]apoCII promoter fragments in the presence or in the absence (control) of STAT1 expression vector. STAT1 overexpression increased the activity of [−545/+18]apoCII promoter in RAW 264.7 cells (∼3.4 fold, p<0.0001) as well as in HepG2 cells (∼1.5 fold, p<0.002). By contrast, the activity of the [−388/+18]apoCII promoter was not increased by STAT1 overexpression, either in macrophages (p>0.5) or in hepatocytes. *Panel C.* RAW 264.7 macrophages were transfected with ME.2[−545/+18]apoCII-luc or with ME.2[−388/+18]apoCII-luc plasmids in the absence (control) or in the presence of STAT1 expression vector. In the presence of ME.2, the activities of [−545/+18] and [−388/+18] promoter fragments were increased by STAT1 overexpression (3.8 fold, p<0.0002 and 2.9 fold, respectively p<0.0002). *Panel D.* DNA pull down assays were performed using different fragments ([−545/+18] and [−388/+18]) of apoCII promoter or oligonucleotides containing the wild type or mutated [−507/−485] region of apoCII promoter, and nuclear extracts obtained from STAT1- overexpressing HepG2 (right) and RAW 264.7 (left) cells. The results showed that the [−545/+18]apoCII promoter, but not [−388/+18] fragment can bind STAT1 proteins (lane 1 and, respectively 2). STAT1 is able to bind the wild type [−507/−485] region of apoCII promoter (lane 4), while STAT1 binding to the mutated region is impaired (lane 5). Negative controls, in which DNAP was performed without DNA, are illustrated in lanes 3 and 6 (no DNA). *Panel E.* ChIP experiments performed using anti-STAT1 antibodies showed that STAT1 is recruited to the [−738/−336]apoCII gene fragment as well as to the [140–440] ME.2 region (lanes 1 and, respectively, 4). No bands were obtained when anti-STAT1 antibodies were omitted in ChIP experiments for apoCII promoter and for ME.2 region (lanes 2 and, respectively, 5). PCR using the input as template and primers for apoCII promoter or ME.2 gave the expected bands (lanes 3 and, respectively, 6). *Panel F.* Schematic representation of STAT1 binding sites on apoCII proximal promoter and ME.2 region.

Next, we searched for STAT1 binding sites on the apoCII proximal and distal regulatory elements. For this purpose, we transiently transfected RAW 264.7 and HepG2 cells with the [−545/+18]apoCII-luc or [−388/+18]apoCII-luc plasmids in the absence (control) or in the presence of a STAT1 expression vector. In RAW 264.7 macrophages as well as in HepG2 cells, STAT1 overexpression increased the activity of the apoCII proximal promoter −545/+18 but not of the apoCII deletion mutant −388/+18 (Fig. 4, Panel A and B respectively). These results suggested that the fragment −545/−388 of the apoCII promoter contains a STAT1 binding site.

When RAW 264.7 macrophages were transfected with the ME.2[−545/+18]apoCII-luc or ME.2[−388/+18]apoCII-luc plasmids in the absence (control) or in the presence of STAT1 expression vector, the activity of both apoCII promoter/enhancer constructs were increased ∼3.8 folds (p<0.0002) and ∼2.9 folds (p<0.0002) respectively by STAT1 overexpression (Fig. 4C). This indicated that the STAT1 binding site that we previously identified in the region 174–182 of ME.2 [23] seems to be essential for the upregulation of the [−388/+18] apoCII promoter fragment which does not contain any STAT1 binding sites.

To investigate the exact position of the STAT1 binding site on the apoCII promoter, we performed an *in silico* analysis using TRANSFAC-MatInspector Software [29]. This analysis predicted a STAT1 binding site in the −500/−493 region of the apoCII promoter. To verify the location of the STAT1 binding site on the apoCII promoter and to test whether this site is functional, we performed DNA pull down (DNAP) assays and chromatin immunoprecipitation (ChIP) experiments. First, we tested the STAT1 binding on the −545/+18 and −388/+18 fragments of the apoCII promoter by DNAP assays using nuclear extracts obtained from human STAT1-overexpressing HepG2 cells. As expected, the whole apoCII proximal promoter −545/+18, but not the −388/+18 fragment could bind STAT1 (Fig. 4D, lanes 1 and 2, respectively). Then, we performed DNAP assays using double stranded oligonucleotides containing the sequence of wild type or mutated −507/−485 region of the apoCII promoter containing the predicted site and nuclear extracts obtained from STAT1-overexpression RAW 264.7 cells. Our data showed that the −507/−485 oligonucleotide was able to bind STAT1 (Fig. 4D, lane 4), while the mutation of five nucleotides in this region impaired STAT1 binding to this fragment (Fig. 4D, lane 5). No bands were detected when DNAP assays were performed without DNA (Fig. 4D, lane 3, and 6 respectively).

To examine the *in vivo* relevance of STAT1 binding to the site described above, ChIP assays were performed. For this purpose, we immunoprecipitated cross-linked chromatin from RAW 264.7 macrophages using anti-STAT1 antibodies and we performed PCR using primers that amplified the fragment 140–440 of ME.2 and the region −738/−336 of the apoCII gene (described in the Table 1). The results of the ChIP experiments performed in RAW 264.7 cells showed that STAT1 was recruited to the apoCII promoter and to the ME.2 as shown in Fig. 4E, lanes 1 and 4, respectively. No binding of STAT1 proteins was observed on the apoCII promoter or ME.2 region when PCR was performed using chromatin that was not immunoprecipitated with the anti-STAT1 antibodies (Fig. 4E, lanes 2 and respectively 5). PCR using the input as template and primers for the apoCII promoter or ME.2 gave the expected bands presented in the Fig. 4E, lanes 3 and 6, respectively.

Taken together, the results of transient transfections, DNAP and ChIP experiments clearly demonstrated the presence of active functional STAT1 binding site in the −500/−493 region of the apoCII promoter. Binding of STAT1 on the apoCII promoter and on the distal regulatory element located on ME.2 (schematically represented in Fig. 4F) have potent stimulatory effects on apoCII gene expression in macrophages.

### STAT1 Cooperates with RXRα to Modulate apoCII Promoter Activity

**Figure 5:**
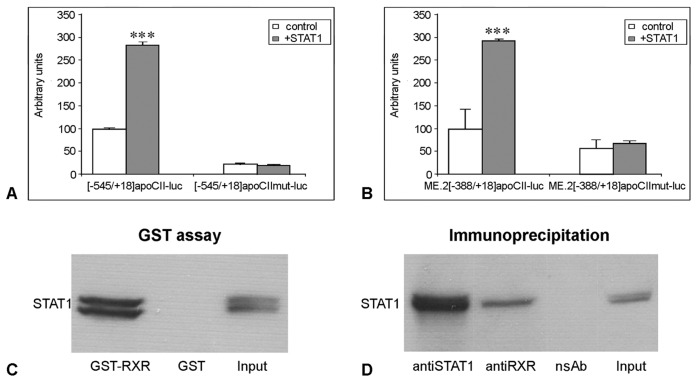
STAT1 interacts with RXRα to modulate apoCII promoter activity. *Panel A, B.* RAW 264.7 macrophages were transiently transfected with [−545/+18]apoCII-luc (A) and ME.2[−388/+18]apoCII-luc (B) or with their corresponding mutants [−545/+18]apoCIImut-luc (A) and ME.2[388/+18]apoCIImut-luc (B) in the presence or in the absence (control) of STAT1 expression vector. The mutation in RXRα/T3Rβ site located at −152/−135 in apoCII promoter impaired the upregulatory effect of STAT1 that could bind to the −500/−493 region of the apoCII promoter (A) or to the 174–182 region of ME.2 (B). *Panel C.* GST-pull down assay performed using cellular extracts from STAT1-overexpressing RAW 264.7 macrophages indicated that STAT1 proteins bind efficiently to the GST-RXRα-beads (lane GST-RXRα), but cannot bind to the GST beads (lane GST); Western blotting for STAT1 using the whole cell extract is illustrated in the lane ‘Input’. *Panel D.* Co-immunoprecipitation experiments were performed using cellular extracts from HEK-293T cells overexpressing both STAT1 and RXRα. The antibodies used for immunoprecipitation are indicated (anti-STAT1 and anti-RXRα). Western blotting of immunoprecipitated proteins done with anti-STAT1 antibodies revealed that STAT1 proteins were efficiently immunoprecipitated by anti-STAT1 antibodies (lane anti-STAT1), but they were also co-immunoprecipitated using anti-RXRα antibodies (lane anti-RXRα). No bands were obtained in the negative control, in which the samples were subjected to immunoprecipitation in the absence of the antibodies (lane NoAb). Western blot for STAT1 using whole cell extract is presented in the lane input.

It was shown previously by our group that the apoCII promoter contains a Hormone Response Element (element CIIC, −152/−35) that binds the orphan receptors ARP-1 and EAR-3 and the ligand-dependent nuclear receptors RXRα and T3Rβ [14]. To test if the RXRα/T3Rβ binding site located in the apoCII promoter is important for STAT1 transactivation, RAW 264.7 macrophages were transiently transfected with the [−545/+18]apoCII-luc and ME.2[−388/+18]apoCII-luc plasmids or with the corresponding plasmids bearing mutations in the RXRα/T3Rβ binding site ([−545/+18]apoCIImut-luc and ME.2[−388/+18]apoCIImut-luc), in the presence or in the absence (control) of a STAT1 expression vector. As expected, the activity of the wild type [−545/+18]apoCII promoter was increased by STAT1 overexpression (Fig. 5A, [−545/+18]apoCII-luc columns). Surprisingly, the activity of the promoter bearing mutation in the RXRα/T3Rβ site was similar in the presence or absence of STAT1 overexpression (p>0.09, Fig. 5A, [−545/+18]apoCIImut-luc columns). These data suggested that the mutation in the RXRα/T3Rβ site located at position −152/−135 of the apoCII promoter abolished the apoCII promoter transactivation by STAT1 via binding to the apoCII promoter. In addition, while the activity of wild type ME.2[−388/+18]apoCII promoter was increased by STAT1 acting on ME.2 (Fig. 5B, ME.2[−388/+18]apoCII-luc columns), no significant difference (p>0.25) between the activity of ME.2[−388/+18]apoCIImut-luc construct in the presence or absence of STAT1 overexpression was noticed (Fig. 5B, ME.2[−388/+18]apoCII mut-luc columns). These data suggested that the mutation in RXRα/T3Rβ site of the apoCII promoter impaired the transactivation of the apoCII promoter by STAT1 via binding to the 174–182 of ME.2.

As control, we tested the capacity of the mutant element [−152/−135] of the apoCII promoter to bind RXRα. Transient transfection experiments showed that the activity of the [−388/+18]apoCII promoter was increased by RA, while the activity of the [−388/+18]apoCII promoter bearing mutations in the RXRα/T3Rβ binding site [−152/−135] could not be increased by RA (data not shown).

Taken together, these results indicated that the mutation in the RXRα/T3Rβ site of the apoCII promoter abrogated the upregulatory effect of STAT1 either via proximal or distal regulatory elements.

### Physical Interactions between STAT1 and RXRα

To test whether STAT1 can physically interact with RXRα, GST pull down assays and co-immunoprecipitations were performed. For GST pull down assays, cellular extracts obtained from STAT1-overexpressing RAW 264.7 macrophages were incubated with GST-RXRα beads and subjected to Western blotting for STAT1. As shown in Fig. 5C, STAT1 efficiently bound to the GST-RXRα-beads (Fig. 5C, lane GST-RXRα) but not to the GST-beads that were used as a negative control (Fig. 5C lane GST). Western blotting for STAT1 using whole cell extract is showed in Fig. 5C, lane Input.

Co-immunoprecipitation of STAT1 and RXRα was performed using cellular extracts obtained from HEK-293T cells overexpressing both STAT1 and RXRα proteins. The samples were immunoprecipated using anti-STAT1 or anti-RXRα antibodies and were subjected to Western blot analysis using rabbit anti-STAT1 antibodies. The results showed that STAT1 was efficiently immunoprecipitated by anti-STAT1 antibodies (Fig. 5D, lane anti-STAT1), but was also co-immunoprecipitated using anti-RXRα antibodies (Fig. 5D, lane anti-RXRα). No bands were obtained in the negative control in which the samples were subjected to co-immunoprecipitation with non-specific antibodies raised in rabbit (Fig. 5D, lane nsAb). Western blot using whole cell extract is presented in Fig. 5D, lane Input.

### Transactivation of the apoCII Promoter by STAT1 is Amplified by RXRα Ligands in Macrophage

**Figure 6:**
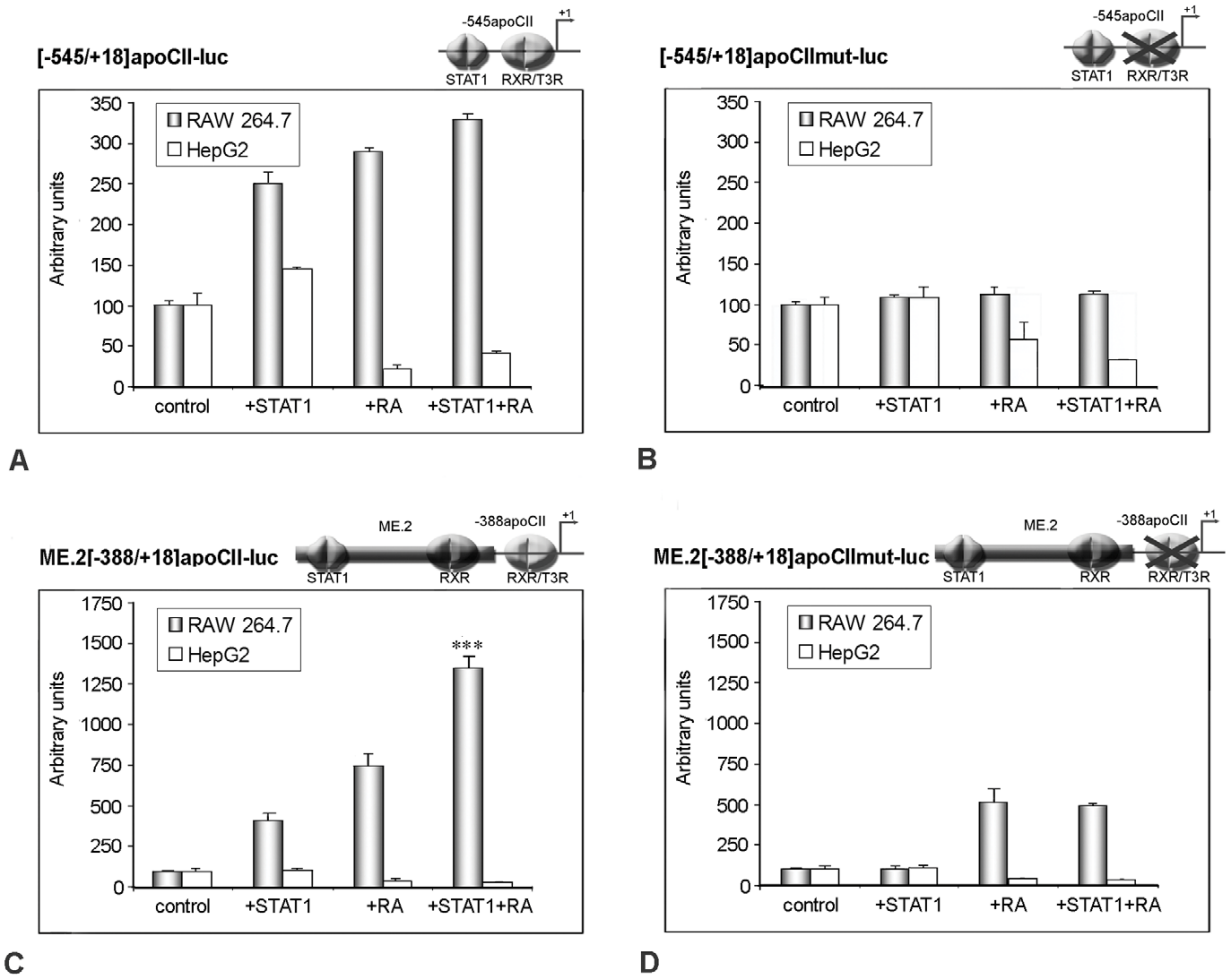
Transactivation of apoCII promoter by STAT1 is amplified by RXRα ligand in macrophages, but not in hepatocytes. *Panel A – D.* RAW 264.7 macrophages and HepG2 hepatocytes were transiently transfected with the constructs [−545/+18]apoCII-luc, [−545/+18]apoCIImut-luc, ME.2[−388/+18]apoCII-luc, and ME.2[−388/+18]apoCIImut-luc under basal condition (control), in the presence of STAT1 overexpression (STAT1), after 9-cis-retinoic acid treatment (RA) or in the presence of both activators (STAT1+RA). The concomitant STAT1 overexpression and RA treatment of RAW 264.7 macrophages enhanced the wild type apoCII promoter at a higher level than each activator alone (Panel A, grey columns). This effect on apoCII promoter was more pronounced when the promoter was under the control of ME.2 (Panel C, grey columns). The activity of the mutant apoCII promoter was not affected by either activator alone (STAT1 or RA) or in combination (Panel B, grey columns). In the presence of ME.2, RA treatment of RAW 264.7 cells increased the activity of the mutant apoCII promoter (due to the RXR binding sites found on ME.2), but STAT1 could not potentiate this enhancement (Panel D, grey columns). By contrast, in HepG2 cells the cumulative effect of STAT1 and RXR was not observed on any constructs employed (white columns).

In order to investigate the role of RXR ligands in the transactivation of the apoCII promoter by STAT1 in macrophages, RAW 264.7 cells were transiently transfected with the following constructs: [−545/+18]apoCII-luc, [−545/+18]apoCII mut-luc, ME.2[−545/+18]apoCII-luc, and ME.2[−545/+18]apoCIImut-luc (Fig. 6A–D). The activity of the above constructs was analyzed under basal condition (Fig. 6, columns ‘control’), in the presence of STAT1 overexpression (Fig. 6, ‘STAT1’ columns), following 9-cis-retinoic acid treatment (Fig. 6, ‘RA’ columns) or in the presence of both activators (Fig. 6, ‘STAT1+RA’ columns). As control, the same experiments were performed in HepG2 cells. The results showed that in macrophages, but not in hepatocytes, the simultaneous STAT1 overexpression and RA treatment enhanced the activity of the wild type [−545/+18]apoCII promoter at a higher level than each activator alone (Fig. 6A, grey columns). In macrophages, the positive effect of simultaneous action of STAT1 and RA was more pronounced on the construct ME.2[−388/+18]apoCII-luc (Fig. 6C, grey columns). These data revealed that STAT1 overexpression and RA treatment exert an additive effect on wild type apoCII promoter activity. They also suggested that, for apoCII transactivation, RXRα interacts stronger with STAT1 bound on ME.2 than with STAT1 bound on the apoCII promoter. The activity of the [−545/+18]apoCII promoter bearing mutations in the RXRα/T3Rβ site was not affected by either STAT1 or RA, nor by their simultaneous presence (Fig. 6B, grey columns). In the presence of ME.2, RA treatment increased the activity of the mutant [−388/+18]apoCII promoter, due to the binding sites found on ME.2, but STAT1 could not potentiate this enhancement (Fig. 6D, grey columns). By contrast, in HepG2 cells the cumulative effect of STAT1 and RXR was not observed on any constructs employed (Fig. 6, white columns).

Taken together, these data indicated that the RXRα/T3Rβ binding site on the apoCII promoter is critical for STAT1-mediated transactivation of this promoter in macrophages.

**Figure 7:**
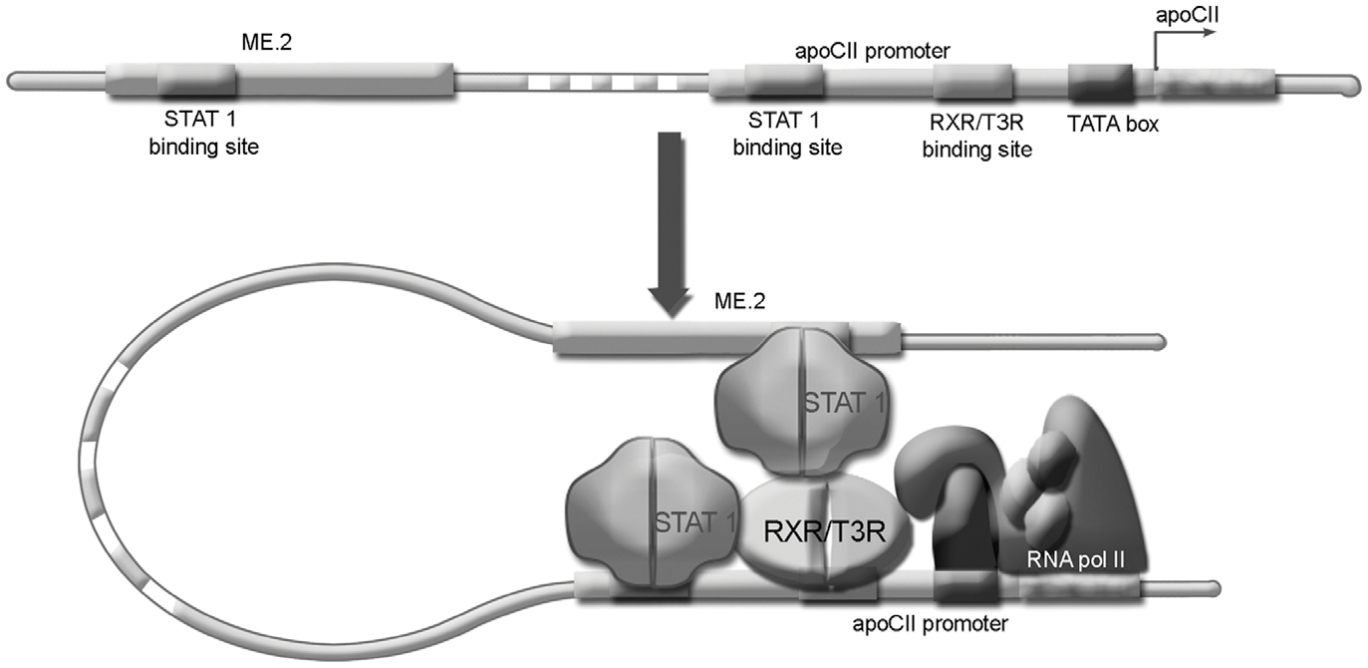
Schematic representation showing STAT1/RXRα interactions in apoCII gene regulation. In macrophages, the ME.2 is brought in the proximity of apoCII promoter. This DNA bending facilitates the interaction of STAT1 attached on its site found on ME.2 with RXRα bound on the apoCII promoter leading to apoCII gene transactivation. A similar mechanism of apoCII transactivation involving interaction of STAT1 with RXRα takes place when STAT1 is attached on its site found on apoCII promoter. After STAT1-RXRα interaction, this complex may cooperate with the basal transcription factors of the initiation transcription complex, thus leading to apoCII gene activation.

## Discussion

The role of apoCII in the catabolism of triglyceride-rich lipoproteins is well established [1]–[2], [10], [30]. ApoCII, at the physiological plasma level, is a specific activator of lipoprotein lipase and plays a central role in regulating the lipoprotein-derived triglyceride hydrolysis. It was demonstrated that deficiency of apoCII or mutations in the apoCII gene is associated with familial hypertriglyceridaemia in humans [31]–[33]. Conversely, it was shown that apoCII overexpression in transgenic mice causes severe hypertriglyceridaemia [34]. Therefore, balanced regulatory mechanisms are important for an appropriate apoCII gene expression. The mechanisms by which the transcription factors that bind to the proximal or distal regulatory elements lead to apoCII gene modulation are not fully elucidated.

In the current study, we uncover the role of the proximal and distal regulatory elements in apoCII gene regulation in macrophages. Data from the literature showed that the apoE/apoCI/apoCI’/apoCIV/apoCII gene cluster contains distal regulatory elements that cooperate with different promoters of the cluster for cell specific expression and for gene modulation. Previous data revealed that in hepatocytes, an interaction between a distal regulatory element (HCR.1) and the proximal apoCII promoter is involved in apoCII gene regulation [13]. For apoE, another gene of the cluster, it was demonstrated that the expression in macrophages is controlled by two homologous regulatory regions, ME.1 and ME.2 [17], [23]. We report here that apoCII promoter activity is enhanced by ME.2 in a macrophage-specific manner (Fig. 1). In addition, 3C experiments demonstrated that, in macrophages, ME.2 physically interacts with apoCII promoter (Fig. 2).

DNase I footprinting analysis using rat liver nuclear extracts identified five protected regions within the [−545/+18]apoCII promoter, as follows: [−497/−462], [−288/−265], [−159/−116], [−102/−81], and [−74/−44] ([13]). In the present work, we focused our study on the 5′- region of the apoCII promoter. We performed an *in silico* analysis [29] and determined a STAT binding site in the −500/−493 region of apoCII promoter. Our previous data showed that differentiation of monocytes into macrophages upon PMA treatment is accompanied by a strong increase in STAT1 protein synthesis. As a response to PMA, STAT1 proteins accumulate; one can assume that STAT1 could be one of the transcription factors that determine apoCII gene expression in monocytes-derived macrophages. Thus, we questioned the role of STAT1 on apoCII gene regulation in macrophages. Our experiments showed that STAT1 overexpression in RAW 264.7 cells was associated with an increase in apoCII mRNA levels compared with control cells while STAT1 inhibition significantly decreased apoCII expression (Fig. 3). These results were similar with those obtained with PMA-activated THP-1 cells, suggesting that the activity of mouse and human apoCII promoters are similarly modulated. The alignment of human ME.2 and mouse ME shows conservation of the STAT1 binding site in the 5′ region of the enhancer. On the human and mouse apoCII promoters, STAT1 and RXR binding sites are present, although they are located at different positions.

Our data revealed the STAT1-dependent transactivation of the apoCII promoter, indicating that there are two biologically active STAT1 binding sites found in the −500/−493 region of the apoCII promoter and in the 174–182 region of ME.2 (Fig. 4). These data demonstrate that, besides the role of the proximal apoCII promoter, the distal regulatory elements of ME.2 may also contribute to the gene regulation, under specific conditions. In addition, distal regulatory elements may modulate various genes found in a cluster. Thus, STAT1 binding site found in 174–182 region of ME.2 is involved in apoCII as well as in apoE gene upregulation (as previously described in [23]).

Next, we tested STAT1 transactivation on apoCII promoter mutated at the RXRα/T3Rβ regulatory element and showed that this mutation abolished STAT1-mediated transactivation of the apoCII promoter (Fig. 5). To explain these intriguing data we proposed two hypotheses: (i) ME.2 could not interact with apoCII promoter when RXRα/T3Rβ binding site was mutated, and (ii) STAT1 should interact with RXRα/T3Rβ in order to transactivate apoCII promoter. We tested the first hypothesis by experiments aiming to determine the capacity of the ME.2 to enhance the wild type and mutated apoCII promoter activity. Thus, we performed transient transfection experiments using plasmids [−388+18]apoCII-luc and [−388+18]apoCIImut-luc or the corresponding constructs in which ME.2 was cloned upstream of the apoCII promoter. The results indicated that ME.2 could interact with the mutated −388/+18 apoCII promoter increasing its activity to a similar degree as the wild type apoCII promoter (data not shown). These data indicated that the mutated form of the promoter retains its ability to interact with ME.2, and do not confirm the first hypothesis.

The second hypothesis that proposes the interaction between RXRα/T3Rβ and STAT1 was tested by GST pull down and co-immunoprecipitation experiments. The results clearly indicated for the first time in the literature that STAT1 can physically interact with RXRα (Fig. 5). Thus, in addition to the known nuclear receptors (RAR, T3Rβ, PPAR, LXR, and FXR) with which RXRα can form heterodimers [35], we clearly established that RXRα interacts with STAT1 transcription factor. Moreover, our data showed that the RXRα/T3Rβ binding site in apoCII promoter is important for STAT1 transactivation via proximal and distal regulatory elements (Fig. 6).

A schematic model for the interaction between the apoCII promoter and ME.2 is shown in Figure 7. In macrophages, ME.2 is brought in the proximity of the apoCII promoter. This DNA bending facilitates the interaction of STAT1 bound on ME.2 with RXRα bound on the apoCII promoter leading to apoCII gene transactivation. A similar mechanism of apoCII transactivation involving interaction of STAT1 with RXRα takes place when STAT1 is attached on the apoCII promoter. Probably due to spatial constrictions, the interaction between STAT1 and RXRα bound on the apoCII promoter is weaker than the long-range interaction of STAT1 bound on the ME.2 and RXRα on the apoCII promoter. This STAT1-RXRα complex may cooperate with the basal transcription factors of the transcription initiation complex, thus leading to apoCII gene activation.

Taken together, our data provide novel mechanisms of apoCII gene regulation involving STAT1 and RXRα transcription factors bound to proximal promoter and distal enhancer elements. In addition, our data reveal that the cooperation between these transcription factors is critical for the proper accomplishment of their regulatory function.
